# Embodiment in a virtual body that speaks produces agency over the speaking but does not necessarily influence subsequent real speaking

**DOI:** 10.1038/s41598-017-14620-5

**Published:** 2017-10-27

**Authors:** Domna Banakou, Mel Slater

**Affiliations:** 1 Event Lab, Department of Clinical Psychology and Psychobiology, Faculty of Psychology, Barcelona, Spain; 2 Institute of Neurosciences, University of Barcelona, 08035 Barcelona, Spain; 3 Institució Catalana de Recerca i Estudis Avançats (ICREA), Passeig Lluís Companys, 23 08010 Barcelona, Spain; 4 Department of Computer Science, University College London, London, WC1E 6BT United Kingdom

## Abstract

Previous results have shown that body ownership, induced through first-person perspective (1PP) over a virtual body (VB) that moves synchronously with real body movements, can lead to illusory agency over VB utterances even though the participant does not speak. It was also found that when participants later speak they follow the fundamental frequency (FF) of the voice of their VB, indicating a new motor plan for speaking. To eliminate the contribution of veridical agency over the VB movements, we conducted a study where we induced body ownership using visuotactile (VT) synchrony rather than visuomotor. Participants saw a life-sized VB from 1PP and reflected in a virtual mirror, that spoke with corresponding lip movements. Half of the 36 experimental participants experienced synchronous (Sync) passive VT on their hands and abdomen, and the other half asynchronous (Async). We found that both VT Sync and Async conditions resulted in a strong subjective illusion of body ownership and agency over the VB, but not, however, changes in voice FF in subsequent speaking. This shows that although illusory agency may be associated with body ownership, a change in motor plan is likely to be a generalisation from veridical agency over whole body movements.

## Introduction

Action control and action perception are two crucial cues of embodied cognition, which significantly contribute to the self-recognition process, and the interaction of the self with the environment. Self-consciousness relies on two components: (a) the experience of oneself as the owner of one’s body (body ownership), and (b) the experience of oneself as the agent of one’s actions (agency)^1^. Previous research has distinguished the sense of agency from the sense of ownership in that the former refers to the subject’s sense of authorship of an action whereas the second includes the subject experiencing the action as pertaining to his or her own body. For example, authorship of moving the body is distinct from the sensation that it is one’s own body that is moving^2,3^. This relationship between agency and ownership has been extensively addressed in the literature with some studies providing evidence that the two factors are independent^4,5^ whereas others support their interaction^6–9^. The sensation of agency has been the subject of significant research in recent years, and self-attribution of actions has been explained by a combination of modalities. These include feed-forward processing^10–12^, intentions prior to action^13^, no alternative explanation for the action result^14^, and a requirement for tight temporal binding between the intention to carry out the action and the resulting sensory consequences^12–15^.

Although empirical evidence shows that the awareness of action and the sense of agency over that action usually go hand by hand, previous results have shown that a sense of agency can be evoked even in the absence of self-executed actions^16–20^. We recently suggested that body-ownership combined with veridical agency over a virtual body might be another cue for experiencing agency over an action not executed by the individual – referred to as *illusory agency* ^16^. Specifically, participants who viewed a virtual body from first-person perspective (1PP) in immersive Virtual Reality (VR), and which moved synchronously with their own real movements, thus providing clear veridical agency, illusorily attributed to themselves the act of their virtual body speaking even though they themselves did not speak (illusory agency). Additionally, they later mimicked the voice of the virtual body, by shifting the fundamental frequency (FF) of their own utterances towards the stimulus voice. We refer to this as a *behavioural after-effect*. Both illusory agency and the behavioural after-effect were extinguished when asynchronous body movements were provided (leaving very little veridical agency except for head movements) and corresponding also to low levels of body-ownership. We suggested that actual agency over virtual body movements, produced by real-time motion capture mapped onto the virtual body, associated with a strong illusion of body ownership later generalised to illusory agency over an action that the virtual body executed but which was not executed by the participant. This was explained in the context of current theories of agency, while taking into account the critical role of ownership over the virtual body. It was proposed that agency over one’s body movements may generalise to other acts carried out by the virtual body itself in the context of body ownership over the virtual body. This could possibly lead to a retrospective intention to act where having experienced the owned virtual body carry out an action, the central nervous system constructs a plan for preparation of future acts of the same type (in that case acts of speaking). This would account for the behavioural after-effect.

In a more recent experimental study^21^, adult participants were embodied in the body of a child, which they experienced from 1PP and with synchronous visuomotor correlations (veridical agency). It found that when their feedback voice (in terms of vocal characteristics) was modified to match that of a child, there was a recalibration of their post-experiment FF towards the signal voice. The effect was not observed when there was incongruence between the feedback voice and the type of the virtual body - that is when participants saw themselves embodied in an adult-looking body, but heard the voice of a child. In that case there was a shift of FF in the opposite direction in order to compensate for the visual-auditory inconsistency.

In an earlier related setup, Kokkinara, *et al*.^17^ showed that participants can have the illusion of agency over walking when they perceived their virtual body to be walking, even though they themselves were seated and only head movements were allowed. Specifically, participants saw a life-sized virtual body spatially coincident with their own from 1PP, or the virtual body from third-person perspective (3PP). After a short period of acclimatisation, the virtual body began to walk. The results showed a strong illusion of body ownership, and illusory agency over the virtual walking, which was supported both by subjective responses and increased levels of physiological arousal while they experienced their virtual bodies to be walking up a virtual hill, but only when perceived from 1PP.

In the new study presented here we were most interested in studying the specific contribution of body ownership to the behavioural after-effect. In particular, we were concerned with whether body ownership *in itself* can account for the after-effect or whether it is necessary that the body ownership be primarily caused by visuomotor synchrony between movements of the participant and movements of the virtual body (always in the context of 1PP over the virtual body). In other words, if there were not the visuomotor contribution to body ownership, would the after-effect still occur?

In order to test this, we aimed at inducing ownership over a virtual body without there being veridical agency with respect to overall body movements (i.e., without synchronous visuomotor – VM - correlations). The hypothesis was that if the previously demonstrated effects were indeed obtained as a result of a new motor plan for speaking, occurring as a result of a generalisation from veridical agency (VM synchrony) over the body to illusory agency over the speaking, then the change in participants’ FF should not occur if body ownership were to be induced without VM synchrony.

## Methods

### Design

**Figure 1:**
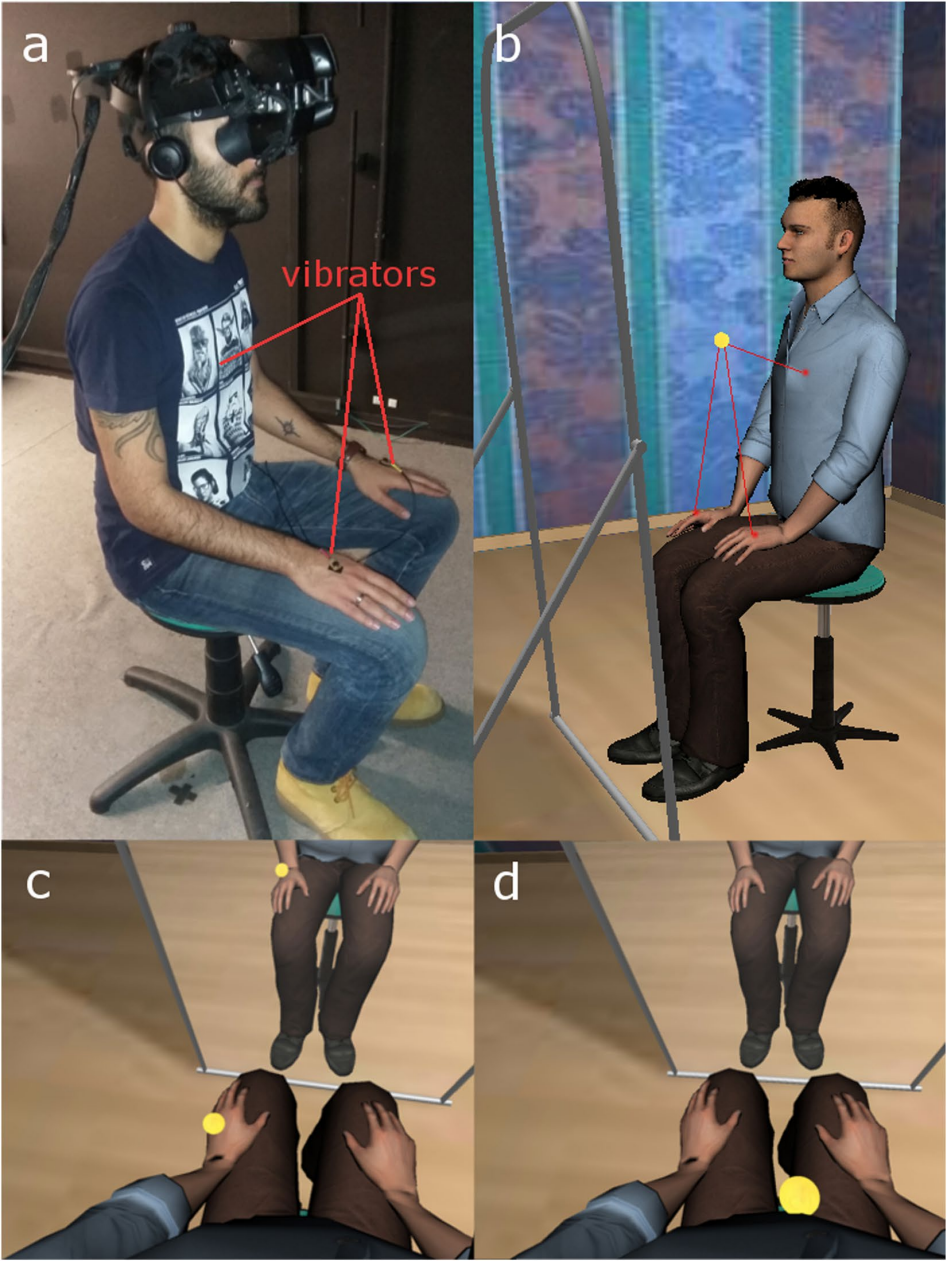
The experimental setup. The body of the participant was substituted by a gender-matched VB, viewed from 1PP, onto which visuotactile stimulation was applied with the help of mechanical vibrators. The body could also be seen as reflected in a virtual mirror. (**a**) Participants wore a HMD with a headset equipped with earphones and a microphone, and a set of mechanical vibrators attached to their hands and abdomen. (**b**) The collocated male VB showing in the red lines the trajectories of the virtual ball. The tapping alternated among the three locations in random order. (**c**,**d**) The male VB seen through the HMD from 1PP directly when looking toward it, and also in the virtual mirror when the virtual ball collided with the hands and abdomen.

We conducted an experiment using a between-groups design, with a single binary factor referred to as ‘Visuotactile’ (VT) with two levels (Async, Sync). Participants were immersed in a VR scenario, where they were provided with a virtual body, seen from a 1PP (Fig. 1). Synchronous or asynchronous passive VT feedback was applied on participants' hands and abdomen with the help of mechanical vibrators. Based on previous results, we expected that the VT Sync condition would result in a stronger illusion of body ownership over the virtual body compared to the Async condition, but not necessarily result in illusory agency^6^, and according to our main hypothesis not result in the behavioural after-effect. Participants were allocated sequentially to one of the two cells of the factorial design in order of attendance to the experiment, with the final numbers as shown in Table 1. There is equal distribution of participants in the cells of the experimental design, and sex balance. An outline of the experiment can be seen in Supplementary Video S1.

**Table 1:** Experimental Design and Distribution of Participants for Experiments 1 (VM) and 2 (VT).

		**Sync**	**Async**
Visuomotor (VM)	Males (n)	6	4
	Mean ± SD Age	23 ± 3.7	22 ± 2.6
	Median Code Previous VR Experience (IQR)	1(0)	1(0)
	Median Code Games (IQR)	2(0)	3(0)
	Females (n)	5	6
	Mean ± SD Age	23 ± 7.1	22 ± 1.7
	Median Code Previous VR Experience (IQR)	1(0)	1(0)
	Median Code Games (IQR)	3(3)	3(1.5)
Visuotactile (VT)	Males (n)	9	9
	Mean ± SD Age	22.6 ± 4.9	22.4 ± 3.17
	Median Code Previous VR Experience (IQR)	2(1.5)	4(5)
	Median Code Games (IQR)	3(2)	3(3.5)
	Females (n)	9	9
	Mean ± SD Age	22.3 ± 3	21.2 ± 3.19
	Median Code Previous VR Experience (IQR)	4(2.5)	3(3)
	Median Code Games (IQR)	2(1)	2(1)

^*^For each case the total number of participants, mean of ages, median and IQR values for participants’ experience in VR and hours of video games are given. Codes refer to a 1–7 Likert scale. For previous VR experience hours spent playing video games 1 means the least and 7 the most.

### Participants

Thirty-six adult male and female healthy participants with correct or corrected vision were recruited by advertisement and email around the campus of University of Barcelona. They were all native Spanish speakers from Spain. They had no prior knowledge of the experiment, and no or little prior experience of virtual reality. The experimental groups were comparable across a number of variables, including previous experience of VR, and time spent playing computer games (Table 1). Each one received 5€ (euros) for participating. The experiment was approved by Comissió Bioètica of Universitat de Barcelona. All participants gave their written informed consent prior to participating to the experiment. The study was performed according to institutional ethics and national standards for the protection of human participants.

### Materials

The experiment was conducted in a VR lab (width: 3.5 m, length: 4.0 m - back wall to curtain - height: 2.5 m). Participants were fitted with a stereo NVIS nVisor SX111 head-mounted display (HMD) (Fig. 1a). This has dual SXGA displays with 76°H × 64°V degrees field of view (FOV) per eye, totalling a wide field-of-view 102° horizontal and 64° vertical, with a resolution of 1280 × 1024 pixels per eye displayed at 60 Hz. Head tracking was performed by a 6-DOF Intersense IS-900 device. Physical touch was delivered via mechanical vibrators connected to a wired Arduino microcontroller (http://www.arduino.cc/), which was attached to their hands and abdomen respectively (Fig. 1a). The location of the vibrators was adjusted for each participant in order to match the contact points of the ball’s trajectory with the virtual body counterparts. The tapping and vibrations alternated among the different locations on the real and virtual bodies in random order, varying in time intervals (2000–7000 milliseconds), intensity (maximum 9600 rpm) and path speed change (6000–15000 ms). For the VT Sync condition, the time delay of the vibrator activation with respect to the virtual collision corresponds to the communication time between the machine running the VR software and the mechanical vibrators, which was negligible (<10 ms). For the VT Async condition, the vibrators were implemented to be randomly activated so that during the visualization of the virtual tapping, touch and collisions were not correlated. Participants were also fitted with a wireless headset, including speakers and a noise-cancelation microphone (Asus HS 1000 W).

Virtual models were created in 3D Studio Max 2010 and Motion Builder 2012 using the RocketBox avatar Library. The virtual environment was implemented using the XVR software platform^22^.

The target words were the same as those used in^16^. They were randomly selected as one and two-syllable Spanish words of consonant-vowel-consonant (CVC) form. Nine words were used in total (casa, mes, sopa, vela, paz, voz, vaso mesa, copa). The two individuals (one male and one female) recruited for their stimulus voices, i.e., the voices of the virtual body, were chosen because the FFs of their voices were higher than that of the average male and female speaker of Spanish (in Spain). The participant was seated in front of a laptop computer (Dell Inspiron Q15r) wearing a wireless headset fitted with speakers and a noise-cancelation microphone (Asus HS 1000 W) to record the BaseF0. The same headset was used during the experimental conditions to both stream the auditory stimulus and record the post-experiment F0 of the participant. The two stimulus voices were recorded using the same recording device (microphone of Asus HS 1000 W), and the appropriate sex-matched voice was played back during the experiment in stereo. The pitch-modulation of the stimulus voice was implemented using the audio editing software Audacity (v. 2.0.3 for windows 7). All material, including software and hardware, was identical to that described in^16^.

The setup and procedures are further illustrated in Fig. 1 and Video S1. Written and informed consent has been given by the persons shown in the images and video to publish these in this online open-access publication.

### Subjective Response Variables

There was a pre-questionnaire to record basic information about the participants such as age, gender, status, and prior experience of VR and computer games. Immediately after the experiment, participants answered a 10-statement post-questionnaire to assess their subjective experience (Table 2). A 7-point scale was used ranging from −3 to 3, (with −3 indicating ‘strongly disagree’ and 3 indicating ‘strongly agree’). The statements were concerned with the strength of body ownership - *VRBody* (the illusion of body ownership), *Mirror* (the illusion of ownership of the body in the mirror) and *Agency* (being the agent of movements of the virtual body), questions relating to the experience of owning the stimulus voice - *VoiceSourceRoom, VoiceSourceHead, OwnVoice, ModifiedVoice, Speaking*, while others served as control questions - *Features* (resemblance of the virtual body to the own body) and *TwoBodies* (the illusion of having two bodies). The voice-related questions were asked at the very end of the experiment, and there was no prior reference to the issue of voice before the end of the experimental process.

**Table 2:** Questionnaire items.

**Variable name**	**Questionnaire statements**
*VRBody*	I felt that the virtual body I saw when looking down at myself was my own body
*Mirror*	I felt that the virtual body I saw when looking at myself in the mirror was my own body
*Features*	I felt that my virtual body resembled my own (real) body in terms of shape, skin tone, or other visual features
*TwoBodies*	I felt as if I had two bodies
*Agency*	I felt that if I moved my real body (arms, hands, legs), the virtual one would also move accordingly
*VoiceSourceRoom*	It felt as if the voice I heard was coming from somewhere in the room
*VoiceSourceHead*	It felt as if the voice I heard was coming from inside my head
*OwnVoice*	It felt as if the voice I heard was my own voice
*ModifiedVoice*	It felt as if the voice I heard was a modified version of my own voice
*Speaking*	It felt as if I was speaking out the words I heard

All questions were scored on a −3 to +3 scale, where −3 meant least and +3 meant most agreement with the statement.

### Vocal production analysis

During the vocal production analysis, we extracted the fundamental frequency (FF) across the 90 trials for each participant before and after the exposure to the virtual environment in order to track the changes in the acoustics of the produced words (45 trials before the stimulus – baseline, and 45 after the stimulus voice). The computer software Praat^23^ was used for analysis of the speech, and was also for reviewing trials from each participant for discontinuities caused by glottal fry. No participants were excluded due to glottal fry.

### Procedures

Participants attended the experiment at pre-arranged times. Upon arriving, they were given an information sheet to read, and after they agreed to continue with the experiment, they were given a consent form to sign. Before the experiment started, participants were seated in front of a laptop fitted with the headset and were instructed to read out in a clear voice 9 target words displayed in sequence. Each word was recorded five times, in random order, using audio editing software, and used as baseline data for later analysis (BaseF0). Next participants were fitted with the HMD, the mechanical vibrators and microcontroller, a pair of headphones, and a microphone. The view seen through the HMD was calibrated using the method described in^24^.

During the first part of the experiment, participants entered a virtual room that included a virtual mirror. The body of the participant was substituted by a sex-matched virtual body, seen from a 1PP. Participants could see the body both by looking directly towards their real body, and also in the virtual mirror. They were seated on a stool in the physical laboratory, and they could also see a collocated virtual body seated on a virtual stool (Fig. 1b–d). They were instructed to stay still during the whole experimental procedure, and that they could only move their heads to look around the virtual room in all directions during the exploration phase. During this visual exploration, participants were asked to state and describe what they saw, to make sure they were paying attention, and that the system was working properly. After the exploration period (2 mins), participants were asked to focus on their virtual body (both hands and abdomen) in the mirror and by looking down, and to avoid looking away in other directions. For the following 5 min they saw a virtual ball bouncing on their virtual body along a pre-recorded path (Fig. 1b–d). Physical touch was delivered via mechanical vibrators attached to their hands and abdomen respectively. It was essential to make sure that participants paid attention, and that the view through the mirror would not confuse them as to which part of the body was being stroked. Hence, they were instructed to alternate their gaze between looking down towards their bodies and in the virtual mirror every time they heard a beeping sound (approximately every 1 min). Then, they were instructed to pay attention to their virtual face in the mirror, and during the following 5 min 21 s the pre-recorded stimulus voice was played through the headphones while the virtual body’s lips moved synchronously with the spoken words. Each word was played back 14 times.

After this stimulus period, the HMD displayed a black background with written instructions to read out loud the nine target words, which appeared in front of them in random order. Each word was recorded 5 times. After removing the HMD they were asked to complete the post-experimental questionnaire. Next they were paid and debriefed. The whole procedure lasted between 20 and 30 minutes. The experimental operator (female) was present throughout the whole experiment.

### Statistical Analysis

All analysis was carried out with Stata 15. Recall that we refer to the factor as Visuotactile (VT) with levels (Async and Sync). The sample size was computed to give a power of 0.95 for the null hypothesis that the FF means would be the same between the two conditions (Async, Sync) with a Type I error of 0.05. The power calculation was based on the data obtained from the study described in^16^. We chose a power of 0.95 (rather than the conventional 0.80) because our main hypothesis was that there would be no difference in FF (the behavioural after-effect) between VT Async and Sync. In the previous work the means and standard deviations of dF = F0 − BaseF0, that is between the fundamental frequency after the manipulation minus before, were 15.00 ± 12.5 for the visuomotor synchrony group and 0.07 ± 11.00 for the visuomotor asynchrony group. Using the Stata 15 function ‘power twomeans’ the required sample size to find such a difference is 36 (18 per group). Eleven per group would be sufficient for a power of 0.8, and 15 per group for a power of 0.9.

### Data Availability

All data generated or analysed during this study are included in this published article (and its Supplementary Information files).

## Results

### Analysis of Questionnaire Responses

**Figure 2:**
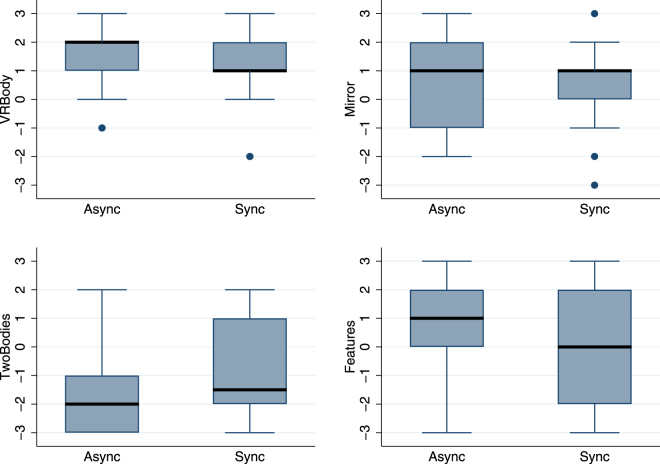
Box plots of body ownership questions by the VT (Async, Sync) conditions. The thick black horizontal lines are the medians, the boxes are the interquartile ranges (IQR), and the whiskers extend from max(smallest value, 25^th^ percentile-1.5 × IQR to min(greatest value, 75^th^ percentile + 1.5*IQR). Values outside this range are shown individually. (**a**) *VRBody* refers to the illusion of ownership over the virtual body seen directly, (**b**) *Mirror* refers to ownership over the body seen in the mirror, (**c**) *Features* refers to the extent to which the virtual body looked like the participant, and (**d**) *TwoBodies* refers to the illusion of having two bodies. See Table 2.

Participants completed the virtual reality experience questionnaire immediately after the experiment (Table 2). Figure 2 shows the median and interquartile ranges of the body ownership questionnaire. It can be seen that the variables *VRBody* and *Mirror* which concern body ownership are high in both conditions (Sync and Async), whereas the control question *TwoBodies* has comparatively low scores, and *Features* somewhat lower in the Sync condition. Hence, in spite of the asynchronous visuotactile stimulation the level of body ownership was high in the Async condition. Ordered logistic regression of each of the questionnaire scores reveals no differences between Async and Sync, with all P > 0.33. Since there are no differences between Async and Sync we can ignore that factor and use Wilcoxon matched pairs signed rank tests to examine differences between the questionnaire scores. *VRBody* is significantly greater than *Mirror* (z = 3.50, P = 0.0005), *Features* (z = 2.83, P = 0.005), *TwoBodies* (z = 4.64, P < 0.00005). *Mirror* is not different from *Features* (z = 1.06, P = 0.288), but significantly greater than *TwoBodies* (z = 3.75, P = 0.0002), and *Features* is significantly greater than *TwoBodies* (z = 3.17, P = 0.0015). It may be surprising that *VRBody* is greater than *Mirror*. Looking in more detail, a pairwise comparison reveals that out of the 36 participants 34 have *VRBody* ≥ *Mirror* of which 16 have *VRBody* > *Mirror*. Looking down towards the body participants would see a trunk, arms and legs, without particularly recognisable features. Looking in the mirror, and being quite close to it, they would see the virtual body’s face, which except by chance would not look like their own.

**Figure 3:**
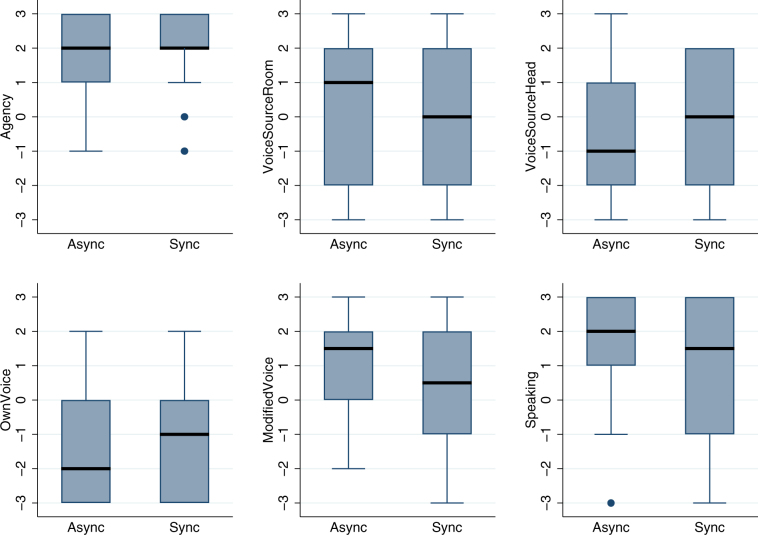
Box plots of agency and speaking by the VT (Async, Sync) conditions. (**a**) *Agency* is the sense of agency over the movements of the virtual body, (**b**) *VoiceSourceRoom* identifies the origin of the voice-higher values mean from the room, (**c**) *VoiceSourceHead* identifies the origin of the voice as from inside the head of the participant, (**d**) *OwnVoice* is the extent to which the voice appeared to be the participants’ own, (**e**) *ModifiedVoice* the extent to which it appeared to be a modified version of their own voices, and (**f**) *Speaking* the extent of agency over the virtual body speaking. See Table 2.

Figure 3 shows the box plots for questions related to agency. Again we find no difference between Sync and Async, suggesting that the conditions that lead to strong body ownership can also result in subjective illusory agency. There was of course some veridical agency since the head movements of the participants were reflected in the head movements of the virtual body, as seen in the mirror. This was the same as in the asynchronous visuomotor condition in^16^ since although in that experiment there was a condition with asynchrony between body movements and virtual body movements, there were always synchronous head movements. Moreover, in the current experiment participants had been instructed not to move their body or limbs, so that they may not have realised that had they moved, the virtual body would not have moved. Participants, as in the earlier experiment^16^, did not talk, so that the *Speaking* response reflects an entirely illusory sense of agency over the speaking. Ordered logistic regression for each of these questionnaire responses shows no difference between Async and Sync, with each P > 0.264. Comparing the scores with the Wilcoxon test as above, *Agency* is greater than all other scores (all P > 0.001) except for *Speaking* (z = 1.63, P = 0.102). *VoiceSourceRoom* is not different from *VoiceSourceHead* (z = 1.32, P = 0.187) and *ModifiedVoice* (z = −0.91, P = 0.363), and is less than *Speaking* (z = −2.53, P = 0.011). *VoiceSourceHead* is greater than *OwnVoice* (z = 2.14, z = 0.032), less than *ModifiedVoice* (z = −2.99, P = 0.003), and less than *Speaking* (z = −3.497, P = 0.0005). *OwnVoice* is less than *ModifiedVoice* (z = −4.37, P < 0.00005), and less than *Speaking* (z = −4.06, P < 0.00005). Finally *ModifedVoice* is not different to *Speaking* (z = −1.16, P = 0.247). The two most interesting responses are *Agency* and *Speaking*. The first indicates that participants had the subjective illusion of authorship over body movements (although there were no body movements except possibly of the head) and the second that the speaking was their own (untrue).

### Vocal Production Analysis

**Figure 4:**
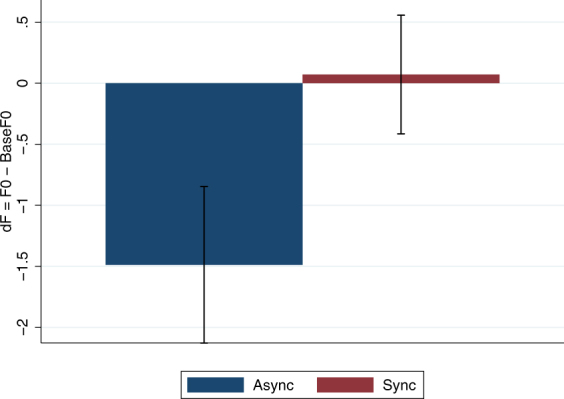
Bar chart showing means and standard errors of dF by the VT (Async, Sync) conditions. The fundamental frequencies of participants’ voices were recorded just before the experiment (BaseF0) and immediately after having been exposed to the stimulus voice (F0).

As in the experiment described in^16^, the FFs of the voices of the participants uttering the same words used in the stimulus were recorded immediately before the experiment (BaseF0). They were recorded again immediately after the stimulation period of the experiment (F0) while still wearing the HMD. The variable of interest is dF = F0 − BaseF0. Figure 4 shows the means and standard errors of dF. This excludes 24 individual dF values with extremely high absolute values of the residual errors after a model fit (with n = 1604). (Whether these are included or not makes no difference to the findings). The VT Async mean is almost the same and negative as that reported for visuomotor async in^16^, and the mean VT Sync value is close to zero. Figure 4, however, does not take into account the repeated values over participants and the words. A mixed effects regression with fixed effect the VT factor and random effects over the individuals, and taking account of the different words, shows little evidence of a difference between the mean dF responses with respect to Async and Sync (z = 1.14, P = 0.256) and the 95% confidence interval for the coefficient of Visuotactile (Async = 0, Sync = 1) is −1.16 to 4.38. The residual errors of the fit are strongly symmetrical about zero following the pattern of a normal distribution, but the distribution does not satisfy the Shapiro-Wilk normality test, the same as was found in^16^.

## Discussion

In Banakou and Slater^16^ we provided evidence suggesting that strong body ownership induced through 1PP combined with visuomotor synchrony between real and virtual body movements can lead to a new motor planning for subsequent actions of the same type as the virtual body - speaking. This was evident through participants producing voice FFs closer to that of the voice of the virtual body. Here we extend these findings, highlighting the importance of veridical agency (i.e., visuomotor synchrony) over the virtual body as an important contributor to this result. We have shown that although body ownership induced through visuotactile stimulation, rather than visuomotor, can still lead to high levels of *subjective agency*, a shift in the FF did not occur in the case of body ownership produced by 1PP and synchronous visuotactile stimulation.

Previously^16^ we argued that these findings might be explained by major theories about the sensation of agency, including the internal forward model and a retrospective intention prior to action that mobilizes the same brain areas as in a prospective intention to act^10,25^. We also discussed how priming could have affected the judgement of agency^26^, although no explicit priming has been included in our studies other than the first utterances of the virtual body, which might be considered as priming for later utterances. Additionally, we addressed the possibility of mimicry^27,28^ that could have arisen between self-movements and the movements of the virtual body, resulting in a possible chameleon effect^29^ that might generalise to the event of speaking. Our results were explained within the framework suggested by Tsakiris, *et al*.^30^ who argued that in the primary motor cortex different movements can overlap in their activations (shared activation between seeing and moving the body and seeing and hearing a talking face). We suggested that participants with visuomotor synchrony continuously activated multiple areas in the motor cortex before and during the talking phase, providing a unified experience with all actions attributed to the self. This is now supported by the current findings, where ownership caused by passive tactile stimulation did not result in a change in voice FF, although it did result in subjective illusory agency. Unlike the primary motor cortex, the primary somatosensory cortex is segmented such that stimulation on one specific point on the body does not generalise beyond the point being stimulated. Here participants in the VT Sync condition experienced synchronous tapping on their hands and chest that occurred only during the exploration phase but not during the speaking experience. Additionally, it is highly unlikely that participants had moved their head during the speaking stimulation, as they were asked to focus on their faces in the mirror, and therefore, there could not have been much activation in the motor cortex during that phase. Hence, there is further evidence suggesting that our earlier findings can be explained as a generalisation of veridical agency over the movements of the virtual body to the behavioural after-effect, and not simply the result of body ownership induced by any method at all, since when there is body ownership but very little veridical agency, the after-effect does not occur. Notice also that although these data do not rule out the possibility that the mean Sync dF > mean Async dF, the mean Sync dF is in any case approximately zero.

With regard to participants’ subjective experiences of ownership and agency over the virtual body, we found in the earlier experiment that synchronous visuomotor, and in this experiment that both visuotactile conditions, give strong effects, and also result in illusory agency over the speaking. Earlier studies comparing the effects of VM and VT synchronous stimulation on body ownership illusions have shown similar levels of ownership for both active movement and passive simulation, either when tested separately^7,31–33^ or in combination^8^. For example, Maselli and Slater^33^ showed that participants report an equally strong illusion for both visuotactile and visuomotor stimulation when they experience a realistic human VB seen from 1PP and which spatially coincides with their real body. Similarly, the authors in^8^ found high ownership scores over a VB for both VM and VT, suggesting, however, that congruent VM stimulation leads to stronger production of the illusion compared to congruent VT stimulation.

Nonetheless, although as found in^16^ visuomotor asynchrony seems to be the most effective in diminishing both subjective ownership and agency over the virtual body, and illusory agency over speaking, the results may seem to be anomalous for the VT asynchronous condition. However, this finding is not surprising in the context of previous studies suggesting that an ownership illusion can be sustained despite asynchronous visuotactile stimulation, provided that congruent visual sensorimotor contingencies are available in the context of 1PP^33–35^. In our experiment, although participants were asked not to move their bodies, they were able to move their heads and look around. In fact, they were asked to first look around and describe the virtual scene, and later on, during the VT stimulation, they were asked to change their gaze between looking directly down to their body or their reflection in the mirror (see Methods for details). Note that during these actions they would not be seeing much of the virtual head movement in the mirror – when looking away of course they would not see the mirror, and when looking directly into the mirror most of the time the head would be stationary. However, this level of agency over the head movements, combined with 1PP and collocation of participants’ real and virtual bodies, appeared to be sufficient to create an ownership illusion, under which visuotactile asynchrony was not influential. In turn, notwithstanding this discrepancy participants also reported a subjective illusion of agency over the whole body, and also illusory agency over the speaking, in both VT Sync and Async conditions. Nonetheless, this did not result in a behavioural after-effect.

In line with our study, participants in^17^ reported a sense of control over the walking movements of their virtual body. This was despite the fact that they did not experience any synchronous visuomotor movements between their real and virtual body, but rather only synchronous head movements, similar to our setup. The authors suggested that their findings were a result of the combination of viewing the walking legs and an intention created by visual flow similar to our everyday walking experience. They argued that since walking is a highly automated motor behaviour, there could be a preparation of the motor commands to walk, which could have failed were participants to be presented with a different, less automated, action. Similar findings were reported by Leonardis, *et al*.^36^ where there was an illusory feeling of walking, but only when visual stimulation was combined with other modalities, such as vestibular and proprioceptive.

Interestingly, participants in the VT Async condition appeared to move their FFs in the opposite direction to the stimulus voice, just as in the VM Async condition as reported in^16^. We discussed in^16^ how following the stimulus voice serves to bring the participant’s voice to agree with that of the external source, whereas compensation works as an error-correction mechanism in order to return the signal closer to that intended by the speaker^37–39^. The current findings further support this idea. Participants in the VT Sync condition did not follow the stimulus voice, and it seems from the results (see Fig. 4) that they did not compensate for this either, but they rather kept it unchanged. Although this is not significantly different from the asynchronous conditions, it could be argued that were there other modalities present during the speaking experience, participants might have exhibited a shifting in the FFs towards the stimulus voice as in the VM Sync condition. In fact, in^16^ half of the participants additionally experienced vibrational feedback on the thyroid cartilage that was synchronised to the syllables of the words being said in order to enhance the sense of speaking. Although VM synchrony was the dominant factor, there was also a significant contribution of the vibrations to the effects. It was argued that the vibrations could have acted as primes since this particular stimulus was always associated with the speaking. This and other modalities that could possibly facilitate illusory agency in the absence of visuomotor correlations are to be further explored in the future, along with neuroimaging studies aiming to understand the underlying neural mechanisms of illusory agency.

Our previous suggestion^16^ was that the illusory agency over the speaking might be accounted for by modified version of the forward model of agency^10–12^, where the intention to act is checked against the prediction of the consequences of the action, and agency occurs when these concur. Since in these experiments the speaking was unexpected there could have been no intention to act, and hence no efference copy or prediction. We argued, however, that the first few words spoken by the virtual body acted as primes, and in the context of body ownership would be taken as self-utterances and a motor plan would be prepared for speaking in the same way. This would then also account for the behavioural after-effect. However, a major motivation for this new experiment was whether this occurred only because of body ownership, or body ownership specifically associated with veridical agency over the whole body as in the first experiment. In the present experiment we do find body ownership and illusory agency over the speaking, but we do not find the behavioural after-effect. Hence, it seems unlikely that the illusory agency is the result of the forward model of agency in the way that we had earlier surmised. Rather we can draw the following conclusions: (a) The factors that give rise to body ownership can also lead to illusory agency. This is conditional on the requirements that if the real body moves as a whole (limbs and trunk) then the virtual body must be moved synchronously, or that the real body does not move and the virtual body also does not move (except for the possibility of head movements which are always synchronous with the virtual body head movements). (b) Thus illusory agency and body ownership go together under these conditions – provided that there is no sensory evidence to the contrary: it is as if the brain hypothesises ‘This is my body, it is talking, therefore I must be talking’. (c) However, the results suggest that preparation of a new motor plan requires strong evidence of actual agency over the body as a whole, which then manifests itself in the behavioural after-effect – in line with Tsakiris, *et al*.^30^. (d) The illusory agency is not caused by the preparation of the new motor plan.

## Electronic supplementary material

Video S1

Supplementary Dataset 1
